# Eradication of Swine Vesicular Disease in Italy

**DOI:** 10.3390/v12111269

**Published:** 2020-11-07

**Authors:** Marco Tamba, Francesco Plasmati, Emiliana Brocchi, Luigi Ruocco

**Affiliations:** 1 Istituto Zooprofilattico Sperimentale della Lombardia e dell’Emilia Romagna, National Reference Centre for Vesicular Diseases, Via Bianchi 9, 25124 Brescia, Italy; emiliana.brocchi@izsler.it; 2 Istituto Zooprofilattico Sperimentale del Mezzogiorno, Epidemiological Veterinary Centre Via Panebianco, 301-87100 Cosenza, Italy; francesco.plasmati@izsmportici.it; 3 Ministry of Health, General Directorate of Animal Health and Veterinary Medicinal Products, Viale Giorgio Ribotta, 5-00144 Roma, Italy; l.ruocco@sanita.it

**Keywords:** swine vesicular disease, eradication, Italy

## Abstract

Swine vesicular disease (SVD) is a contagious viral disease of pigs clinically indistinguishable from other vesicular diseases, such as foot and mouth disease, vesicular stomatitis, vesicular exanthema of swine, and idiopathic vesicular disease. In Italy, where SVD was first reported in 1966, an eradication program started in 1995. The program, updated in 2008, was based on regionalization, complete control on pig movements, improvement of pig farms biosecurity, appropriate cleansing and disinfection procedures of vehicles approved for pig transportation, and a testing program using both serological and virological assays. In cases of confirmed SVD virus infection a stamping-out policy was applied. In the period 2009 to 2019, between 300,000 and 400,000 pigs were serologically tested each year. The last SVD outbreak was notified in 2015, and the last seropositive pig was detected in 2017. SVD surveillance is still ongoing and no proof of virus activity has been detected so far. All available data support the complete SVD virus eradication from the Italian pig industry.

## 1. Introduction

### 1.1. Characteristics of Swine Vesicular Disease

Swine vesicular disease (SVD) is a contagious viral disease of pigs caused by a member of the *Enterovirus* genus of the Picornaviridae family. The disease agent (SVDV) was identified as a porcine variant of human coxsackievirus B5 [1] and all isolates are classified in a single serotype, with four distinguishable antigenic/genomic variants [2]. The genome of SVDV consists of a single positive strand of messenger-active RNA comprising a 5′ non-coding region (NCR) followed by an open reading frame encoding the polyprotein precursor to the structural (P1) and the non-structural (P2 and P3) proteins, a short 3′ NCR, and finally a poly(A) tract at the 3′ terminus [1].

The first outbreak of the disease was reported in Italy in 1966 [3]. Europe has experienced several epidemics in the past, but during the last ten years SVD has been notified only in Italy. The disease is likely to be present in various parts of eastern Asia where the last reported case of SVD was in Taiwan in 2000 [4].

The clinical signs of SVD are identical to those of foot and mouth disease (FMD), as well as of other vesicular diseases, such as vesicular stomatitis, vesicular exanthema of swine, and idiopathic vesicular disease caused by Senecavirus A [5]. The severity of the lesions depends on the virus strain and the type of housing, with less severe lesions in pigs kept on straw bedding rather than on concrete [1]. The disease is generally mild and mortality is negligible, with recent outbreaks reporting a shift towards subclinical disease courses in infected pigs [6]. All infected pigs, including subclinical animals, shed the virus with feces, contaminating the surrounding environment [4].

SVDV is stable in pig carcasses and pork products for months—more than 300 days in hams and at least 200 days in dry salami, sausages, and casings [7]. Despite the mildness of the disease and the negligible impact on pig production, SVDV has historically been considered a hazard by countries, such as Australia and United States of America when importing live pigs or pork products or by-products.

### 1.2. Swine Vesicular Disease Epidemiology and Keys for Control

Natural SVDV infection has only been reported in swine. SVDV is spread primarily by contact with an infected animal. Infected pigs excrete the virus from nose, mouth, and feces in large amounts before the appearance of clinical signs. Most of the virus is produced in the first week after infection and does not normally persist in infected animals for more than two weeks. On the other hand, excretion with feces continues for longer than three months after infection has been reported [1]. SVDV excretion in feces could be ‘reactivated’ for a short period of time in pigs from which SVDV can no longer be identified, when animals undergo to the physiological stress of mixing. Persistent infection is possible but is probably a rare occurrence with limited significance for the disease epidemiology [8].

SVDV is resistant to routinely-used disinfectants and is relatively stable over a wide range of pH, from 2 to 12, depending on temperature and time [7]. The epidemiology of SVD is mostly related to the extraordinary stability of the virus in the environment. Contact with an environment contaminated with SVDV has been demonstrated to be equally as infectious as direct inoculation or contact with SVDV-infected pigs [9]. Airborne spread is not a route of SVDV transmission. Even on infected premises, spread from one pen to another may not happen in the absence of a common open drainage system, or of frequent movement of pigs between pens. Therefore, SVD could be regarded as a “pen disease” [1]. Nevertheless in a densely-populated small area of Lombardy, Italy (more than 3000 pigs per km^2^) proximity to a previous outbreak was noted as a risk factor for infection, and it was necessary to depopulate a group of pig farms to achieve SVDV eradication [10].

In Italy outbreak investigations have associated SVDV infection with introduction of infected pigs, introduction (into the farm) of inadequately-disinfected vehicles contaminated with SVDV being used to transport infected pigs, or the persistence of the virus on farms following depopulation and inadequate cleansing and disinfection [6,10]. Due to the SVD epidemiology, control measures to achieve eradication of the virus have to be based on: (a) complete control on pig movements, (b) appropriate cleansing and disinfection procedures of vehicles approved for pig transportation, (c) improvement of pig farms biosecurity, and (d) appropriate cleansing and disinfection procedures of infected farms after depopulation. Moreover, due to frequent cases of subclinical infection an active surveillance program based on serological and virological testing is needed [10].

With the aim to update the current status of SVD in Italy; in this paper we report the results of the national surveillance and eradication program carried out in the last eleven years (2009–2019).

## 2. Materials and Methods

### 2.1. Surveillance and Eradication Scheme in Italy

In Italy SVD has been a notifiable disease since 1973. Disease control measures are currently established in the EU by Council Directive 92/119/EEC, Annex II [11]; in the case of confirmed SVD infection a stamping-out policy is required, together with the establishment of protection and surveillance zones in which pig movements are restricted or banned.

A national surveillance and eradication program started in 1995, with the aim of achieving eradication by means of an SVD health certification scheme of pig farms. Within the program, for a holding to be declared as SVD-free, it must be sampled on two different occasions, at a 28 to 40 day interval, and all the serological tests must score negative. A region can be declared SVD-free when 99% of pig farms in the territory have been declared free from SVD. All control actions were performed by the Official Veterinary Services. Details of this program have been described elsewhere [6].

To avoid spread of the disease in the rest of the European Union (EU), a specific regulation (Dec. 2005/779/EC) “concerning animal health protection measures against swine vesicular disease in Italy” was laid down. Pigs in regions not declared as SVD-free should not be dispatched to other member states; pigs in regions declared free from swine vesicular disease should only be dispatched from SVD-free holdings [12].

Equally the United States of America (US) import pork meat and short-seasoned pork products only from regions they have been recognized as SVD-free [13].

After a major epidemic occurred in Lombardy, northern Italy, in 2008 the national eradication program has been revised. In the new program additional activities were planned, in particular:

- Fattening farms were included in the program;
- A higher sensitivity of the surveillance system was obtained by increasing the sample size in the tested farms (Table 1);
- Specific biosecurity requirements were provided for dealers and breeding and fattening farms;
- Dealers’ premises were progressively switched to a special type of fattening farm, called high-turnover operations (HTO), where a monthly serological and virological check was carried out.

The last measure is important, because by the Italian law a dealer can only hold the animals for less than 30 days, while a farmer can hold them for more than 30 days. As a consequence, in the HTO all pig batches are potentially controlled for SVD through serological and virological tests.

Finally in 2011, after a further SVD epidemic occurred in a SVD-free region bordering an SVD-not-free region, Italy set up a special task force composed of experts from the Ministry of Health and from the National Reference Centre for Vesicular Diseases (NRL). The task force provided training for the official veterinary services, supported the regional authorities in outbreak investigations and in planning additional measures to be applied only in SVD-not-free regions. Starting from 2011 in the region of Campania, and from 2015 in Calabria, the following measures were added:

- Filling of a ‘trip and disinfection diary’ for each lorry approved for pig transportation;
- Farms not fulfilling biosecurity requirements were excluded from the market;
- Killing on the spot and destruction without compensation of all pigs of unidentified origin;
- Killing on the spot and destruction without compensation of all pigs in seropositive farms not fulfilling biosecurity requirements.

### 2.2. Diagnostic Tools

In Italy diagnostic procedures for SVD are carried out in compliance with the indications laid down at European Community level by the Commission Decision 2000/428/EC. This regulation establishes diagnostic procedures and sampling methods and criteria for the evaluation of laboratory test results for the confirmation and differential diagnosis of swine vesicular disease [14]. In Italy, the blood samples collected for SVD surveillance are tested at 10 regional laboratories coordinated by the NRL, using the same monoclonal antibody-based competitive ELISA (c-ELISA) recognized by the World Organisation for Animal Health (OIE) as the reference screening test [15,16]. All positive samples are confirmed by the NRL, using the virus neutralization test (VNT) carried out as previously reported [15] and extensively described in the OIE Manual [16]. These tests performed in series have a specificity close to 99.9%. In addition, VNT-positive sera are also submitted to an in-house isotype-specific ELISA to identify the classes of IgM and IgG of anti-SVDV immunoglobulins [15]. False positive reactors, also called singleton reactors, can be discriminated by a combination of VNT, c-ELISA, and isotype-specific ELISA [14,17]. Investigation of singleton reactors demonstrated that: (1) the VNT titer is generally low, (2) virus isolation in feces is negative, (3) seroconversion is never observed in pen-mates, (4) only a small proportion of reactors is still positive at a second sampling (about 7 days later), and (5) only IgM isotype is responsible for the positive reaction [1,14,17].

Details on virological testing procedures have already been described by Bellini et al. [6]. Briefly, virological tests are carried out at the NRL. For virological testing, a reverse transcriptase polymerase chain reaction (RT-PCR) targeting the SVDV 3D region is performed as a screening test [16], and virus isolation is carried out only on RT-PCR-positive samples.

### 2.3. Investigation on Positive Animals Substantiating the Freedom of Disease

A pig having a VNT titer equal to or higher than 1:256 is classified as positive. Accordingly, a herd presenting at least one seropositive pig, is considered as positive. Each seropositive pig is further investigated to determine whether it gave a false or true positive result following the procedure provided by the European legislation [14]. The investigation always includes a rechecking of the positive animals, the serological checking of a sample of in-contact pen-mates (P = 50%; CI 95%), and the verification of possible contacts with other SVD-seropositive or infected farms. Seropositive pigs without a link with positive farms and fulfilling the five criteria above described by De Clercq [17] for singleton reactors, are classified as false positive.

## 3. Results

As a result of the adoption of the new eradication program in 2008, the remaining four SVD-not-free regions gradually achieved the SVD-free status, based on EU legislation. The first region was Sicily (2008), then Abruzzo (2009), followed several years later by Campania (2017). The last region declared as SVD-free was the region of Calabria in 2019 (December 2019/470/EU). Together with this declaration, Decision 2005/779/CE was repealed and pig movement restrictions were removed in the whole country [18].

**Figure 1:**
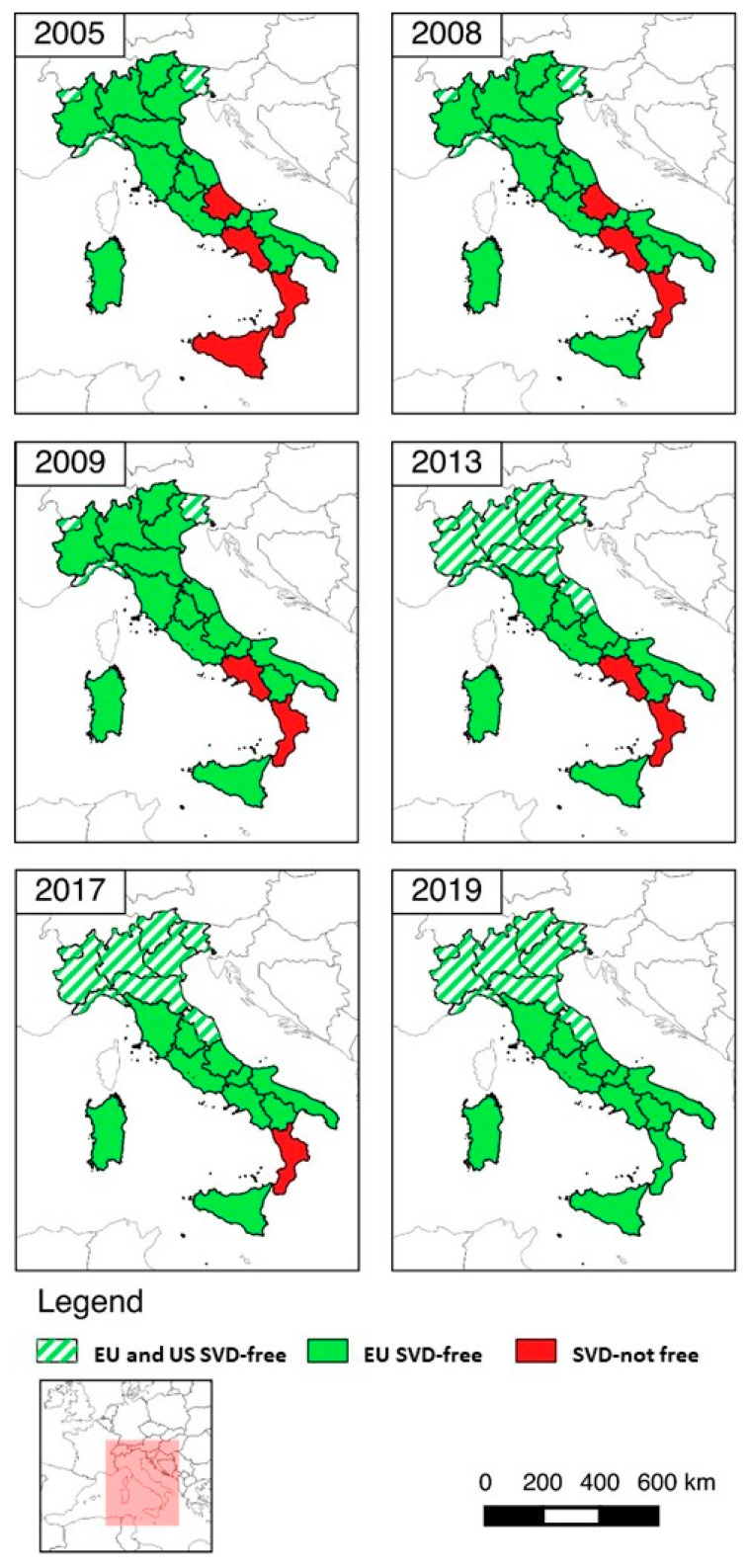
Trend of SVD status of Italian regions, 2005–2019.

In the meantime, in 2013 the US recognized another seven regions (Piedmont, Lombardy, Emilia-Romagna, Veneto, Marche, Autonomous Provinces of Trento, and Bolzano) as SVD-free territories (Figure 1) [13]. These regions rear more than 85% of all Italian pigs.

In the last ten years on average more than 16,500 farms have been tested each year (Table 2). Since 2011 SVD outbreaks have been notified only in SVD-not-free regions or in bordering SVD-free regions of southern Italy (Molise, 2011–2012; Basilicata, 2014). The last SVD outbreak was notified in 2015 in Calabria; the last seropositive farm in the same region was also recorded in 2017.

Serological checks have involved on average more than 60% of the pig industry, and between 300,000 and 400,000 pigs have been sampled each year (Table 3). Incidence of seropositive pigs progressively decreased, but it dramatically dropped after the application of the extraordinary measures in the region of Campania in 2015.

## 4. Discussion

SVD is an important swine disease affecting pigs and, as a result, pork-product exchanges. Italy has a pig industry focused on the production and export of valuable pork products (hams, salami, etc.), and to avoid sanitary barriers towards these products, official veterinary services are continuously requested by risk assessors of importing countries to supply reliable data, possibly substantiating freedom from the disease.

Generally speaking, achievement of disease freedom is possible when there is no clinical, epidemiological, or any other evidence of disease or agent of disease presence in a given period of time within a given geographical area [19]. In Italy the last SVD outbreak was notified in 2015. Moreover, to prove freedom from disease, the official veterinary services have in place a surveillance system where any suspicious sign of disease activity is reported and possible evidence of transmission is investigated by sampling a statistically-selected proportion of the pig population. In either case, any suspicion of disease is followed by quarantine, confirmatory diagnostic procedures, and any necessary disease control activities [19].

Actually, SVD is not included in the OIE terrestrial code, but in the past the disease was listed, and a country could be considered free from SVD when it could demonstrate that the disease has not been present for at least the past two years. This period could be reduced to nine months for countries, such as Italy, in which a stamping-out policy was practiced [20].

In addition to a stamping-out policy, active surveillance programs and control of pig movements are considered the main tools for SVDV eradication. Thanks to the first program (1995–2008), in northern and central Italy, where commercial pig farms are prevalent, eradication of SVDV was achieved in a few years through intensive serological campaigns involving the breeding farms only. In southern Italy more difficulties were encountered because of the presence of thousands of small (1–3 pigs) fattening holdings intended for self-consumption. In such a scenario, pig dealers, in order to provide for their clients, have created a network for the illegal movement of pigs of the desired weight, that also involves fattening farms. This illegal trade allowed several incursions of SVDV in the free regions, probably by means of contaminated lorries [6].

Four out of twenty-one regions were still not free at the time of the second eradication program (2008) which was meant to correct the weaknesses of the previous one. More precisely, increased farm biosecurity has avoided further SVDV incursions in SVD-free regions, and currently cleansing and disinfection of lorries entering the farm is always controlled directly by the farmers.

Inclusion of fattening farms in the program has allowed virus eradication from an additional two regions (Sicily and Abruzzo), but SVD outbreaks still occurred in the remaining SVD-not-free regions and bordering free areas until extraordinary measures hampering illegal pig movements were adopted in 2015. Only thanks to those measures was it possible to also eradicate SVDV in a couple of years from the last two not-free regions (Campania and Calabria).

The last seropositive result related to SVDV infection was detected in 2017. However SVD surveillance is still ongoing in Italy to fulfill the requests of countries importing pork and pork products, and no proof of virus activity has been detected so far. All available data support the complete SVDV eradication from the Italian pig industry as of today.

## Figures and Tables

**Table 1:** Swine Vesicular Disease Surveillance Scheme in Italy, 2008–2019.

SVD Status	Type of Pig Farm:	Farrow-to-Feeder	Farrow-to-Finish	Dealers’ Premises/High Turnover Operations	Fattening Farms
SVD-free regions	Farms under program:	All	All	All	Risk-based sampling (P = 3%; CI = 95%) ^1^
	Sampling in selected farms:	P = 10%; CI = 95% ^2^	P = 10%; CI = 95% ^2^	P = 5%; CI = 95% ^3^	P = 5%; CI = 95% ^4^
	Sampling frequency:	Twice a year	Yearly	Monthly	Twice a year
SVD-not-free regions	Farms under program:	All	All	All	All
	Sampling in selected farms:	P = 10%; CI 95% ^2^	P = 10%; CI= 95% ^2^	P = 5%; CI = 95% ^3^	P = 5%; CI = 95% ^4^
	Sampling frequency:	Twice a year	Yearly	Monthly	Twice a year

Note: ^1^ P: expected prevalence; CI: confidence intervals; sampling of a maximum of 100 farms selected at regional level among those presenting risk factors for SVD (i.e., trading with regions not yet SVD-free, low biosecurity, large size, etc.); ^2^ random sampling of a maximum of 30 breeders (boars, sows, or gilts) distributed among all the pens of the farm; ^3^ random sampling of a maximum of 59 pigs distributed among all the pens of the farm; and ^4^ random sampling of a maximum of 59 fattening pigs distributed among all the pens of the farm and different classes of age.

**Table 2:** Surveillance of SVD in Italy—Serological checks in pig farms by SVD status of the region, 2009–2019.

SVD Status	Year	2009	2010	2011	2012	2013	2014	2015	2016	2017	2018	2019
EU and US SVD-free regions	No. of regions	3	3	3	3	10	10	10	10	10	10	10
	No. of tested farms	244	141	245	237	4106	3685	3942	3620	3610	3438	3464
	No. of seropositive farms				1	22	20	17	27	23	37	40
	No. of true positive farms											
	No. of SVD outbreaks											
EU SVD-free regions	No. of regions	15	16	16	16	9	9	9	9	10	10	11
	No. of tested farms	11,766	12,155	11,860	18,879	19,164	13,675	11,284	9208	9859	11,129	9698
	No. of seropositive farms	104	87	104	68	24	12	10	33	28	33	34
	No. of true positive farms	16	6	15	15	3	2					
	No. of SVD outbreaks	4	2	1	6		2					
SVD-not-free regions	No. of regions	3	2	2	2	2	2	2	2	1	1	
	No. of tested farms	4097	4698	2321	1652	1414	1350	1322	1186	448	442	
	No. of seropositive farms	160	42	85	24	29	32	31	26	5	2	
	No. of true positive farms	139	28	75	16	9	6	18	3	1		
	No. of SVD outbreaks	14	2	24	1	1	3	1				
Italy (all regions)	No. of regions	21	21	21	21	21	21	21	21	21	21	21
	No. of tested farms	16,107	16,994	14,426	20,768	24,684	18,710	16,548	14,014	13,917	15,009	13,162
	No. of seropositive farms	264	129	189	93	75	64	58	86	56	72	74
	No. of true positive farms	155	34	90	31	12	8	18	3	1	0	0
	No. of SVD outbreaks	18	4	25	7	1	5	1	0	0	0	0

Note: EU and US SVD-free regions—regions of Italy declared as free from SVD by the United States Department of Agriculture and the European Commission, EU SVD-free regions—regions of Italy declared as free from SVD by the European Commission, and EU not-free regions—regions of Italy not declared as free from SVD by the European Commission.

**Table 3:** Surveillance of SVD in Italy—Serological checks in pigs, 2009–2019.

Year	Number of Pigs in Italy	Number of Pigs Under the Program	Number of Tested Pigs	Number of Seropositive Pigs	Number of True-Positive Pigs	Indicators	
						% Coverage	Incidence TP/10,000
2009	9,677,126	6,826,154	622,333	885	725	9.1%	11.65
2010	9,547,187	4,763,761	368,615	339	281	7.7%	7.62
2011	9,756,036	5,977,542	379,239	554	346	6.3%	9.12
2012	9,286,297	5,834,350	367,759	399	163	6.3%	4.43
2013	9,050,564	5,357,856	435,629	164	128	8.1%	2.94
2014	8,985,076	5,982,167	412,813	146	119	6.9%	2.88
2015	9,192,821	6,145,170	353,230	152	118	5.7%	3.34
2016	8,944,171	5,870,865	377,398	108	4	6.4%	0.11
2017	9,022,003	5,337,385	362,103	75	9	6.8%	0.25
2018	8,883,908	5,469,305	318,334	94	0	5.8%	0.00
2019	8,612,627	5,498,865	321,581	89	0	5.8%	0.00
